# Accrual-Monitoring Practices for Various Disease Trials among AACI Member Cancer Centers

**DOI:** 10.3390/clinpract12050072

**Published:** 2022-08-31

**Authors:** Zachary T. Elliott, Zachary Goldberg, Ramez Philips, Jennifer M. Johnson, Margaret T. Kasner, William K. Kelly, Sarah Osipowicz, Rachael Dampman, Joseph M. Curry

**Affiliations:** 1 Department of Otolaryngology–Head and Neck Surgery, Thomas Jefferson University, Philadelphia, PA 19144, USA; 2 Department of Medical Oncology, Philadelphia, Thomas Jefferson University, Philadelphia, PA 19144, USA; 3 Sidney Kimmel Cancer Center Protocol Review Committee, Thomas Jefferson University, Philadelphia, PA 19144, USA

**Keywords:** rare diseases, clinical trials, accrual and monitoring

## Abstract

Progress in the management of rare diseases, including rare cancers, is dependent upon clinical trials; however, as many as 32% of rare-disease trials go uncompleted or unpublished due to insufficient accrual. Monitoring practices may differ between institutions. We sought to survey the regulatory standards for various trial types among major U.S. cancer centers. A 10-question survey was designed using Qualtrics assessment software. The survey was sent via email to an internal server of member institutions of the Association of American Cancer Institutes (AACI). Of 103 AACI centers, 31% completed the survey (*n* = 32). Respondents differed in their definitions of a rare disease, minimum expectations for rare tumor studies, and frequency of accrual monitoring by their institutional Protocol Review and Monitoring Committee. Seventy-three percent of respondents did not close trials based on low accrual. Strategies to optimize accrual included investigator incentives for high accrual and penalties for low accrual in 37% and 13% of respondents, respectively.

## 1. Introduction

The definition of a rare disease varies, but the most widely used is the European (EOMC) definition of rare cancer, which is an incidence of <6/100,000 persons per year [1]. However, many institutions have also adopted the National Cancer Institute’s (NCI) definition of a rare disease, defined as a disease affecting <15/100,000 persons per year, with over 7000 diseases meeting this status [2]. The number of individuals with rare diseases may appear relatively small; however, an estimated 30 million people (10% of the U.S. population) have a rare disease [2,3,4,5,6,7]. These statistics will continue to increase. Previously common diseases are now being subclassified based on deep genomic sequencing, with each new disease subclass potentially having unique treatment modalities and responses. This stratification of previously common diseases subsequently leads to a smaller number of subjects in each new disease subclassification [8,9]. Currently, 270 new rare diseases are being identified each year, now accounting for 22% of all cancer diagnoses, which is higher than any single common cancer [1,6].

Little progress has been made in improving the prognoses of rare cancers. This is in contrast to more common diseases, which have seen an overall improvement in prognosis over the same period [1]. Part of this challenge is due to the difficulty in accruing patients to rare-disease trials, resulting in an estimated 25–32% of rare-disease clinical trials being terminated due to low accrual [10,11]. Low accrual not only threatens the successful completion of clinical trials, but also creates significant financial pressures for cancer centers, as valuable resources are utilized to keep complex, low-accruing trials operational. This is a challenge for rare-disease trials that take place in centers without specific expertise in the disease of interest. Many hurdles must be overcome when conducting a rare-disease clinical trial, at the patient, interventional, institutional, and policy levels, due to the changing landscape of treatment options and disease populations [4]. One of the key issues in rare-disease trials is linked to the challenge of achieving evidence of better outcomes in a small population [3]. Accrual into rare-disease randomized controlled trials is difficult and infeasible in many instances. Behera et al. describe a hypothetical example of a trial for indolent T-cell LGL leukemia: “439 patients would be needed to conduct a study to demonstrate a 50% relative risk reduction of death with a total trial duration of 5 years. However, considering the usual 5% patient enrollment into cancer trials, and a disease prevalence of 160 patients per year, it would take up to 55 years to enroll the required number of patients” [12]. Similar recruitment challenges and small sample sizes have led to the development of specialized study designs and biostatistical techniques designed to maximize data from small numbers of subjects [13].

The NCI’s Cancer Center Support Grant requires that NCI-designated cancer centers establish a Protocol Review and Monitoring Committee (PRMC). This committee must scientifically review all trial protocol at onset and continuously to ensure adequate progress and accrual. However, there are no formal guidelines for what constitutes adequate progress or minimum accrual expectations for various study types. We sought to assess minimum accrual expectations across U.S. cancer centers to understand accrual monitoring practices for rare-disease trials and other various categories of studies.

## 2. Materials and Methods

A 10-question survey (Table 1) was designed using Qualtrics assessment software (Salt Lake City, UT, USA). The survey was designed to allow respondents to choose binomial responses and/or leave free-text responses for most questions. A free response format was implemented, given the likelihood of substantial variability and nuances between institutions.

The survey was sent via email to an internal server of the Association of American Cancer Institutes (AACI). The AACI comprises 103 of the leading academic and freestanding cancer research centers in North America. The survey was available for three weeks, with a reminder being sent before closing. Additionally, the Google search engine was queried on 22 February 2022 using search terms “PRMC Charter” and “Protocol Review Committee Charter.” The first 50 results of each search term were reviewed and summarized to find available clinical trial-monitoring charters and policies.

## 3. Results

Of the 103 AACI centers, 32 (31%) completed the survey. There was 1 duplicate respondent from the same institution. Responses to the survey were collected between 10 November 2021 and 23 December 2021. For Question #1, “no expectation for accrual” was the most common response for Rare Tumor Trials and Rare Molecular Subtype Trials. However, for Phase I, Industry-Sponsored, Investigator-Initiated (Same Center), Investigator-Initiated (Another Center), and NCTN/National studies, “50% of target” was the most selected response for accrual expectations (Table 2).

Responses for question #2 were highly individualized, only relative to each respondent’s institution and are, therefore, not reported here. For Question #3, the frequency of the accrual monitoring of studies varied by survey respondent, with over half reporting monitoring at least once every 6 months (Table 3).

For Question #4, 18 respondents (72%) said they did not close trials, including rare tumor studies, that did not meet minimum accrual expectations, while 7 (28%) reported they did (Table 4).

For Question #5, 93.8% of respondents did not limit the number of rare tumor trials. A total of 3.1% of respondents limited the number of rare tumor trials, and 3.1% were unsure. For Question #6, a rare disease was defined differently by survey respondents according to different annual incidence rates or annual prevalence (Table 5).

A total of 22% of respondents had multiple definitions of a rare disease, which allowed for the definitions to be met without meeting the required annual prevalence/incidence. For Question #7, only 9% penalized trials with low accrual. For Question #8, 66% of respondents reported not incentivizing trial accrual, while 19% reported different methods of incentivizing investigators to choose appropriate methods for their patient population to encourage the accrual of patients. These methods include recognition (most popular), increased resource allocation or preference for future trials, and the allocation of credits to investigators for conference registration or travel. Question #9 elaborated on responses from prior questions. The results of question #10 are not reported to institutional identity; however, the sample of institutions represents programs from across the U.S. A total of 11 institutional PRMC charters were identified online (Table 6).

These publicly available charters provided little insight regarding respective policies and procedures, as many did not have specific requirements outlined across all trial types.

## 4. Discussion

These data suggest that clinical trial portfolio-management techniques differ across AACI member institutions. Interestingly, the definition of what constitutes a rare disease varied across respondents. Eight institutions (26.7%) adopted the definition of a rare disease as one with an incidence rate of 15/100,000 per year (consistent with the NCI definition of a rare disease). This is more than double some of the definitions adopted by other institutions that responded to the survey. Additionally, as common cancers are reclassified according to molecular subtypes, previously common cancers are reclassified as diseases occurring at smaller incidences. This reclassification of cancers and the multiple definitions of rarity applied to them creates moving accrual targets for investigators and regulatory committees alike.

Operating trials under a definition of a higher prevalence or incidence would allow for more diseases to be considered rare by institutions (e.g., adopting a definition of incidence 15/100,000 versus 6/100,000). As reported in this survey, few centers had explicit monitoring policies for rare disease and rare-molecular-subtype trials. Using definitions that correlate with a higher incidence or prevalence would allow more diseases to be considered to be rare and to subsequently fall under softer guidelines granted to rare-disease trials. Our survey demonstrated that many institutions provide minimal accrual requirement exemptions (50%) and rare-disease trial closure exemptions (50%). Additionally, PRMC accrual requirements for rare-disease trials were lower compared to non-rare-disease clinical trials. While more laxity is evident for a rare-disease trial, the frequency of PRMC accrual monitoring still occurs at intervals of at least 3 months or less for 60% of respondents.

While different expectations for accrual may be needed for these trial types, investigator-focused incentives may also be employed to ensure that trial accrual does not stagnate, and resources are not wasted. Examples of investigator-focused incentivizing reported in the survey included quarterly/annual recognition and higher prioritization for future trials when specified accrual expectations were surpassed. Some institutions reported monetary awards that could be allocated towards academic seminars and conferences. The widespread adoption of similar programs could help boost accrual numbers and prevent economic waste for uncommon trial types.

Resource allocation is a major consideration when designing and conducting rare-disease trials. Starting and completing a clinical trial is a significant financial undertaking, with the cost of bringing a new drug to the market lying between USD161 million and USD2 billion [14]. Depending on the field of research, the costs of phase 1, 2, and 3 clinical trials conducted in the United States are USD1.4–6.6, 7.0–19.6, and 11.5–52.9 million, respectively [14]. The termination of trials secondary to low accrual represents a significant loss of invested capital. Over 25% of rare disease trials were terminated between 2016 and 2020 due to low accrual rates [11]. The total cost of clinical trials is expected to reach 68.9 billion dollars globally by 2025. Approximately USD491 million of this capital will be lost each year from rare-disease trials closing due to accrual issues alone [11,15,16]. Reshaping the accrual expectation for rare-disease trials could mitigate the loss of substantial amounts of money by allowing them to continue with lower accrual expectations.

The combined goals of investigators and PRMC members alike should be focused on the initiation, continuation, and completion of rare-disease trials to promote needed breakthroughs for these diseases. The differences and difficulties of conducting a rare-disease trial compared to a non-rare-disease trial must be understood by both parties. As demonstrated by the sampled institutions in this survey, rare-disease trials are considered and managed differently from their non-rare-disease counterparts. Rare-disease trial management may also benefit from focusing on each rare-disease trial individually, with considerations of how the rare disease impacts both the population and the design of the specific trial. Ultimately, clear and thorough communication between investigators and their institution’s PRMC may be necessary for them to understand expectations and for the management committee to understand the challenges within each trial. Institutions that currently embrace stricter guidelines may consider and adopt the more flexible practices implemented by the responders of this survey. Conversely, investigators can select protocols that increase the accrual and impact of active trials. Techniques employed to increase the continuity, impact, and completion of rare-disease trials exist in the areas of accrual methods, statistical analysis, and trial design.

### 4.1. Accrual

Difficulties in acquiring subjects for clinical trials secondary to small patient populations distributed over large geographic regions is a unique challenge encountered in the research of a rare disease [7]. To address this issue, patient-advocacy groups (PAGs) were created. PAGs often aid in subject acquisition, research funding, administration of patient assistance programs, and guide communication between patients and physicians. Additionally, the patients themselves and their support organizations are becoming well-informed sources for patient recruitment. The National Organization for Rare Disorders (NORD) also helps with subject recruitment, primarily by employing clinical broadcasts that notify subjects of opportunities to participate in clinical studies. PAGs are also highly effective in acquiring fundraising support for the study of rare diseases. Networking between international PAGs is increasing accrual into new rare-disease clinical trials. The number of rare-disease patients in any one geographical location may be small. In these situations, international networks can help identify trials outside of limited locales [2,13,17]. A survey conducted in 2016 by the Rare Disease Clinical Research Network (RDCRN) found that PAGs helped enroll nearly half of the subjects in trials conducted by PAG members [6,13]. Engagement with PAGs may be a strategy to increase accrual and meet the expectations imposed by institutional PRMCs.

### 4.2. Trial Design

Several solutions for alternative study designs have been proposed for the purpose of improving patient enrollment [18]. Some of these methods may be disease-dependent but can be considered in the implementation for rare-disease trials. For example, trials that guarantee all patients receive an intervention may improve the recruitment rates for patients with rare diseases and limited options for treatment [19]. Another method includes employing crossover trials where patients are randomized to one specific treatment with a time-limited effect and then to no treatment at another time [19]. Other strategies include maximizing the total number of outcomes to reduce the trial’s sample size. Increasing the length of follow-ups may potentially allow more events or outcomes to be captured among a smaller number of participants. However, this strategy would certainly increase the cost of already-difficult-to-fund rare-disease trials. Repeatedly measuring the same outcome or event or using a combined single endpoint composite outcome has been suggested to increase outcomes without increasing the cost of the trials. Similarly, surrogate endpoints or primarily biomarkers may be used in the place of hard outcomes. Surrogate endpoints are predictive of hard outcomes of interest, allowing for measurement before patients are lost to follow-up for hard clinical outcomes. Even a trial with a small sample size could have values measured for the surrogate biomarker of patients, and the possibility of missing hard outcomes to loss of follow-up would be abated [20,21]. Outcomes may also be measured as continuous variables instead of binary outcomes. Maximizing the number of outcomes using surrogate endpoints or implementing continuous outcomes can potentially decrease the total number of subjects required for analysis, while maintaining adequate statistical efficiency. Finally, an adaptive randomization design can be used to lower sample-size requirements. The probability of being randomized to an intervention during the enrollment period for trials increases with adaptive randomization. Overall trial enrollment is reduced with adaptive randomization, while imbalances in baseline covariates among treatment groups are minimized. Additionally, the number of patients partitioned to the hypothetically more effective treatment group is increased [21].

### 4.3. Statistical Considerations

Deviating from the statistical norms may help decrease the required sample sizes for rare-disease trials. For example, investigators could reduce the precision of effect estimation on the outcome of interest. Confidence intervals are associated with the statistical significance of an outcome and are dependent on the trial’s sample size. However, the sample size of a trial could technically be reduced with the compromise of a reduction in the precision of the effect estimation (e.g., using a sample size corresponding to 85–90% confidence interval instead of the stand 95%) [20]. There is also rationale for conducting studies that have subject-size constraints with a power of less than 80%. However, due to the unacceptably high risk of not achieving statistical significance, study sponsors rarely go below 80% power. Additionally, significance achieved in a trial with low power is often regarded as unconvincing [22]. However, since the selection of certain study endpoints corresponds to the required size of the cohort, selecting endpoints that favor smaller cohort sizes can be advantageous. Continuous endpoints typically require a smaller sample size than discrete/binary endpoints, allowing for smaller sample sizes. Additionally, increasing the time for the length of follow-up can allow for a reduction in cohort size, as measures collected at the additional visits make up for the smaller size [20,23].

## 5. Conclusions

Rare cancers continue to have poor median survival rates despite comprising over 1/5 of cancer diagnoses in the United States. Progress in the management of these diseases requires insights gained from clinical trials, but accrual issues hinder their completion. This study utilized a survey to identify strategies employed among US cancer centers to manage different trial types, including rare-disease and rare-molecular studies. Respondents differed in their definitions of a rare disease as well as expectations for rare-tumor studies and the management of low trial accrual. Rare-cancer trials may benefit from a refined focus on each trial individually and the consideration of alternative designs that improve accrual and increase patient and physician acceptability. Future research should investigate opportunities for trial improvement made possible by collaboration and the sharing of management approaches across AACI institutions.

## Figures and Tables

**Table 1:** Survey questions.

The Sidney Kimmel Cancer Center at Thomas Jefferson University is seeking to identify best practices around managing clinical trial portfolios and specifically how best to manage studies with slow and/or low accrual. We appreciate you taking the time to complete this questionnaire.
What are your Protocol Review and Monitoring Committee’s (PRMC) minimum accrual expectations for each of the following categories of studies: Phase 1 Studies: _____ Rare Tumor Studies: _____ Rare Molecule Subtype Studies: _____ Industry Sponsored Studies: _____ Investigator Initiated Studies (From You Center): _____ Investigator Initiated Studies (From You Center): _____ NCTN/National Studies: _____
2. What is your PRMC’s process for warning investigators about studies that do not meet minimum accrual expectations? Over what period of time are these warnings issued?_____
3. How frequently does your PRMC monitor accrual? Annually (select) Twice per year (select) Other Please Specify: _____
4. Will your PRMC close all types of trials that do not meet minimum accrual expectations? Yes (select) No, please explain: _____
5. Does your cancer center limit the number of rare tumor trials? If yes, is this done in total for the cancer center, or is the limit set per disease group? _____
6. What is your center’s definition of a rare disease? _____
7. Does your cancer center penalize trials with low accrual? If yes, how? _____
8. Does your cancer center incentivize trial accrual? If yes, how? _____
9. Is there anything else about your PRMC’s minimum accrual expectations or accrual monitoring process that you would like to share? _____
10. If you would like a summary of all responses to this questionnaire, please share your contact information: Name: _____ Email: _____ Cancer Center: _____

**Table 2:** PRMC minimum accrual expectations by study category.

	Phase 1	Rare Tumor	Rare Molecular Subtypes	Industry Sponsored	Investigator Initiated (Same Center)	Investigator Initiated (Another Center)	NCTN/National
50% of Target	9	4	5	11	12	11	1
40% of Target	1	–	–	1	1	1	1
25% of Target	1	–	–	–	1	–	–
5 Per Year	1	–	–	3	1	1	1
4 Per Year	1	–	–	2	1	2	1
3 Per Year	1	–	–	1	1	1	1
2 Per Year	2	–	1	1	1	1	2
1 Per Year	1	5	7	–	1	1	2
1 Every 7 Years	–	1	1	–	–	–	–
1 Every 5 Years	1	–	–	1	1	1	1
1 Every 2 Years	–	1	1	–	–	–	–
1 Every 6 Months	1	1	–	2	1	2	2
Variable Target	5	4	4	5	6	6	5
No Expectations	7	14	13	4	4	4	4
No Response	1	2	2	1	1	1	1
Total	30	30	30	30	30	30	30

**Table 3:** Frequency of PRMC accrual monitoring.

Answer	Number	%
Every 12 Months	8	25.0%
Every 6 Months	12	37.5%
Every 3 Months	2	6.3%
Every Month	6	18.8%
Varied	4	12.5%
Total	32	100%

**Table 4:** Trial types that were exempt from closure.

Answer	Number	%
Pediatric Population	4	57%
2. PI is a national PI	2	29%
3. Intention-to-treat studies will remain open and ‘rare-cancer types’ if a disease group is accruing into other studies.	1	14%

**Table 5:** Definition of rare disease by annual incidence rate * or annual incidence **.

Answer	Number	%
15/100,000/year *	9	28.1%
6/100,000/year *	14	43.8%
3/100,000/year *	2	6.3%
<200,000 **	2	6.3%
<10,000 **	2	6.3%
<5000 **	1	3.1%
No Response	2	6.3%
Total	32	100%

**Table 6:** Minimum accrual expectations detailed in PRMC charters and policies found via web query of PRMC charters available online. A total of 11 online charters were identified.

Accrual:	Phase 1	Rare Disease	Rare Molecular Subtype	Cooperative	Investigator Initiated Studies
At Least 1 Annually	–	2	–	2	–
At Least 2 Annually	2	–	–	–	–
25% of Annual Goal	1	–	–	1	1
33% of Projected Goal	–	–	–	–	1
50% of Projected Goal	–	–	–	–	2
PRMC Discretion	2	1	–	–	–
Exempt	–	2	1	–	–
Not Outlined	5	5	9	–	–
Not Mentioned	1	1	1	8	7
Total	11	11	11	11	11

## Data Availability

Not applicable.
